# The role of NGOs’ service delivery experience in developing relevant research agendas: experience and challenges among NGOs in Malawi

**DOI:** 10.1186/s12961-017-0199-3

**Published:** 2017-05-04

**Authors:** Kate Gooding

**Affiliations:** 1 Malawi-Liverpool-Wellcome Trust Clinical Research Programme, Blantyre, Malawi; 2 Liverpool School of Tropical Medicine, Liverpool, UK

**Keywords:** Non-governmental organisations, Civil society, Research agendas, Research prioritisation, Malawi

## Abstract

**Background:**

There has been growing interest in the contribution of non-governmental organisations (NGOs) to international health research. One strength that NGOs may bring to research involves the potential value of service delivery experience for indicating relevant research questions, namely through their involvement in service delivery, NGO staff may be aware of frontline knowledge gaps, allowing these staff to identify questions that lead to research with immediate relevance. However, there is little empirical evidence on research agendas within NGOs to assess whether their service delivery experience does lead to relevant research or conditions that affect this. This article examines the identification and selection of research questions within NGOs to explore the role of their service delivery experience in generating relevant research agendas.

**Methods:**

The article reports comparative case study research on four NGOs in Malawi, including two international and two Malawian organisations. Each NGO conducts research and undertakes service delivery and advocacy. Data collection included interviews, focus groups, observation and document review. Analysis involved thematic coding and use of diagrams.

**Results:**

The case NGOs’ experiences suggest that using service delivery to identify research questions does not always match NGOs’ aims or capacities, and does not guarantee relevance. First, NGOs do not want to rely only on service delivery when developing research agendas; they consider other criteria and additional sources of ideas when selecting questions they see as relevant. Second, service delivery staff are not always well-placed to identify research topics; indeed, involvement in hectic, target-driven service delivery can hinder input to research agendas. Third, NGOs’ ability to pursue questions inspired by service delivery depends on control over their research agendas; relationships with external actors and financial autonomy affect NGOs’ capacity to undertake the research they see as relevant. Finally, the perceived relevance of research findings varies between audiences and depends on more than the research question.

**Conclusions:**

The findings suggest limits to the value and feasibility of a research agenda based on service delivery experience. Based on the analysis, the conclusion outlines strategies to support an effective role for NGOs’ service delivery experience in development of research agendas.

## Background

The need for relevance is widely stressed in discussions on international health research [1–4]. Much research in developing countries does not fit local priorities, reducing its impact, leaving gaps in the information needed for policy and practice, and presenting an unethical waste of resources [5, 6]. Non-governmental organisations (NGOs) may be well-positioned to support more relevant research due to their involvement in service delivery. Through their service delivery, NGO staff might identify research questions that respond to frontline knowledge gaps, leading to research with immediate relevance. As stated by Zachariah et al. [7] when describing one NGO’s approach, “*research questions are generated by identifying the constraints and challenges of implementing each programme's medical activities. The answers provided to these questions should then have direct, practical relevance to solving the identified constraints and challenges*”. Similarly, Kidwell-Drake et al. [8] highlight the benefit of an NGO consortium for producing a “*field-driven research agenda*”, suggesting that “*NGO staff should be well informed of the obstacles to implementation*”, leading to research focused on “*concerns that are relevant to the end users*”.

This potential value of NGOs’ service delivery as a source of relevant questions is one rationale behind growing calls for NGO involvement in research prioritisation [9–14]. The role of service delivery in generating questions is also given as a reason for academics to work with NGOs [12, 15–18], and as a reason for NGOs to pursue research. For example, explaining why NGOs should become involved in research, one NGO network policy suggests that “*close to the difficulties and problems of translating the ‘big’ approaches into daily life,* [NGOs] *have a huge insight and are in an excellent position to ask the ‘right’ questions*” [19]. Not only can useful research questions come from NGOs’ service delivery, some suggest research questions “*generated from within programmes*” are an “*essential element*” of NGO research [7].

However, there is limited empirical research on NGOs to assess whether or when their service delivery experience does lead to relevant research. Literature on NGO involvement in research is growing, but detailed studies are scarce and there have been calls for further work [7, 10, 20–26]. Within existing literature, the role of service delivery as a source of research questions is often noted briefly, with little information on the way relationships between NGOs’ service delivery and research agendas operate in practice. Further evidence on conditions affecting the contribution of service delivery could help NGOs and their research partners to draw on service delivery effectively when identifying research questions.

This article examines experience in four NGOs in Malawi to explore the role of their service delivery experience in generating relevant research, examining whether NGOs’ research agendas are based on their service delivery experience and whether questions based on service delivery lead to relevant research.

Relevance is a key issue in discussions about health research in Malawi. Government strategies emphasise the need to increase relevance, noting a dominance of externally-driven research that does not fit to national priorities [27, 28]. A National Health Research Agenda was developed to indicate research needs [29–31] and research priorities have also been identified for specific health issues [32].

NGOs are important health service providers in Malawi [27]; they have been part of initiatives to prioritise health research [29, 32], and NGOs are recognised as stakeholders within national research policies [28, 31]. Although research remains a marginal activity within the NGO sector, some NGOs are significant research producers and there is growing interest in research among NGOs in Malawi.

The concept of relevance is ambiguous and contested. Academic discussions within international health research and beyond highlight the difficulties of judging and defining relevance, and the political and economic factors that affect these definitions [33–36]. Articles on the contribution of NGO service delivery to research tend to understand relevance in instrumental terms [37, 38], seeing research as designed to have immediate application in policy or practice [e.g. 7, 8]. In this perspective, research should address problems identified by implementers, meeting NGOs’ “*need to find out ways to overcome technical and organisational constraints*” [19]. Research is relevant if it can improve existing interventions, support new interventions, or help influence policy [7]. Thus, research is “*valued only to the extent that it produces results that can be acted upon or put into practice*” [39]. Some academics and civil society activists question this instrumental interpretation of relevance as a goal for research. They see a focus on use in policy or practice as potentially damaging space for reflection, critique and independence [40–43], and argue that producing “*strategic, hegemony-challenging knowledge*” can be more important than applied, problem-solving research [42]. More critical research that questions dominant health and development frameworks or highlights wider structures that affect programme outcomes could well be considered relevant to NGOs’ work; indeed, the kind of research that is relevant for NGOs has been debated between development NGOs and academics [33]. The present article, however, focuses on an instrumental definition of relevance, both because this is the understanding of those who suggest that involvement in service delivery can support relevance, and because, as explained later, this instrumental approach reflects the case NGOs’ research interests.

## Methods

This was a qualitative study using comparative case studies.

### Setting

The four case study organisations have been given pseudonyms. Two cases are Malawian NGOs. Flint is large by the standards of Malawian NGOs and works on issues affecting women and young people, including sexual health. Clay is medium-sized and undertakes community mobilisation in several sectors, including HIV and gender. The other cases are international NGOs, with headquarters in high-income countries. Marl is very large and focuses on health service delivery, while Chalk is medium-sized and works in sectors such as HIV, nutrition, sanitation and livelihoods.

All four NGOs undertake research alongside service delivery and advocacy. Their research approaches and capacities vary. Flint conduct situation analyses to support specific projects or influence wider policy, and assessments of government services to support advocacy. Studies often use mixed methods, such as surveys, interviews and focus groups. A few staff have short-term experience of university or NGO research, or short-term research training, but there are no staff with dedicated time for research. Clay undertake participatory action research to stimulate behaviour change and mixed-methods situation analyses. They have a full-time research manager, and several staff have long-term action research experience. Marl has an established record of research aimed at internal, national and international audiences. Most studies either test new interventions or assess existing activities, using quantitative trials or qualitative approaches. The two full-time research staff both have several years of experience in Northern universities, and Marl recruit additional research assistants as needed. Chalk’s research is growing, and involves assessing NGO and government interventions, field testing new technologies and conducting situation analyses. There is a research manager with masters-level research training, whose time is split between research and other programme support, and several other staff have some university or NGO research experience.

### Data collection

Data were collected during 6 months’ fieldwork in Malawi, followed by further email, Skype and telephone conversations to check interpretations and fill information gaps. In-depth interviews were conducted with 33 purposively selected staff or former staff in the four case NGOs, including staff leading on research, directors and those involved in service delivery and advocacy. An additional eight repeat interviews were conducted with key staff members. Most interviews lasted 1–1.5 hours. Frequent informal conversations with these and other staff provided further information. In three NGOs (flint, chalk and clay), focus group discussions of approximately 2 hours were held with between four and five staff. Most focus group participants were also interviewed individually, but group discussions complemented interview data by providing opportunities for interaction.

Observation of NGO activities provided further information on research processes and organisational contexts. Approximately 5 weeks were spent working from Flint’s office. In other NGOs, logistics restricted observation to attending relevant meetings and some informal ‘hanging around’.

Document review provided contextual information on each NGO, and included material identified from websites and provided by staff, for example, organisational strategies and research reports.

To provide information on NGOs’ relationships with other actors and external perceptions of NGOs’ research, and to indicate whether experiences from the case NGOs were shared more widely, 11 additional interviews were conducted with donors, government officials, academics and staff from two ‘mini’ case NGOs.

Topic guides were specific to each interview or focus group depending on participants’ roles and issues emerging from previous data collection, but covered areas such as research involvement, identification of research questions, interactions with external stakeholders, variations between research projects, and views on completed research. Most interviews and focus groups were audio-recorded and transcribed verbatim. When recording was impractical, detailed notes were taken and then expanded.

### Analysis

Analysis employed thematic coding by hand and using NVivo, and matrices and network diagrams to explore relationships between themes and variations between and within organisations. Conflicting evidence and alternative interpretations were examined to check and refine interpretations.

### Ethical approval and consent

The study was approved by the University of Leeds ethics committee in the UK (HSLTLM/11/004) and the National Commission for Science and Technology (RTT/2/20) and Centre for Social Research (CSR/11/11/05) in Malawi. All participants provided oral informed consent, based on provision of information sheets and discussion about study processes, confidentiality, risks and benefits. Oral consent, supported by ongoing, open communication, was more comfortable for participants than written consent, and better suited to fieldwork involving repeated and sometimes informal interaction with participants [44, 45].

## Results

The case NGOs’ experiences highlighted four steps that affect the link between service delivery and relevant research, namely whether NGOs want to use their service delivery experience to identify research questions, whether staff involved in service delivery suggest research questions, whether NGOs can pursue research questions they identify and consider relevant, and whether questions based on service delivery lead to relevant research findings. These four steps are considered in turn.

### NGOs’ interest in using service delivery to identify research questions

When identifying research topics they want to follow, the case NGOs do not just consider their service delivery experience; they also consider other sources of ideas and additional criteria unrelated to service delivery.

In relation to other sources, some research questions selected as priorities are identified by NGO staff through involvement in non-service delivery activities such as advocacy. For example, a Marl investigation into medical training capacity was prompted by proposed government funding cuts, which they learnt about through advocacy networks. In other cases, research questions are suggested by external partners. Universities regularly approach Chalk with ideas for collaborative research. Ideas that fit Chalk’s objectives and can be accommodated are usually taken forward, and Chalk staff often view the resulting findings positively. One example is research with a university to pilot an energy technology linked to existing household products promoted by Chalk. The resulting data “*have been useful for programming*” (programme director), providing information on the household technology that has informed Chalk’s work. In particular, the findings challenged common assumptions about some benefits of the household products, which altered Chalk’s messages to communities when promoting these products. They also provided additional information on resources required for use of the products within households, helping project planning. The new energy technology is also considered viable, and Chalk are expanding use of this technology in their programmes, indicating the potential relevance of externally-initiated research.

When research questions do originate from service delivery, the NGOs’ decisions on relevance involve additional criteria. This multiplicity of criteria is seen in comments about Marl’s annual research planning:

“*We’re thinking about all the different policies that are being done in the country and what we’re seeing in the hospital and what the published research has found and trying to come up with what we think makes sense for research in this setting and is in line with Marl’s philosophy*.” (research coordinator)

Service delivery is one source of ideas, as indicated by reference to issues seen in the hospital. However, research prioritisation also considers whether topics are appropriate for Marl, existing research, and national policy developments.

On the first of these criteria, Marl choose research questions that fit organisational strengths. In particular, Marl feel they are best-placed to examine practical implementation: “*we don’t want to be a clinical randomised control trial, that’s not what the field setting is about*” (research coordinator). Similarly, Chalk see their niche as examining social and cultural rather than technical issues, and they select research topics that fit this: “*the social focus is very important.* […] *This is the area where we can add some value, not just number crunching*” (director). Following this principle, a study on an agricultural technology examined cultural factors that affect implementation rather than technical features (such as construction). Similarly, potential research on malaria was dismissed because the research manager felt Chalk lacked skills to investigate clinical topics and the university was better placed to pursue such research. Although Chalk see technical and clinical research as relevant for their work, other organisations conduct such research, and Chalk’s proximity to implementation and relationship with communities allows them to investigate social and cultural issues that other research organisations might miss: “*we don’t do technical research as there is lots of this that you can find through Google. It’s more the social or anthropological side, people’s perceptions*” (research manager).

On the second aspect of existing research, Marl and Chalk check whether proposed research questions are already answered to avoid wasting resources and to assess whether the research would expand current knowledge and thus generate wider interest. Service delivery staff are sometimes unaware of existing evidence on questions they face in the field, so “*they come up with a topic but then you Google it and find it’s already been done*” (chalk research manager). The third aspect of policy developments also indicates likely wider interest. While some questions come directly from policy networks as above, Chalk and Marl also consider whether questions identified through service delivery fit national policy discussions. For example, Marl started research on a health volunteer project partly because they needed to know whether it worked, but also because lessons could inform a forthcoming government policy. As well as considering these criteria internally, the NGOs sometimes seek feedback from external partners. For example, Chalk discuss potential topics with their networks to see whether “*others are already doing that or there’s no interest in it*” (research manager), and they consult government partners to see whether proposed topics fit policy priorities.

The specific criteria and sources of ideas valued in the case NGOs reflect their research aims. For example, the interest in advocacy networks as a source of ideas, and in policy relevance and wider interest as criteria for topic selection, applied with research aimed at external influence. These sources and criteria may be less relevant when research aims only at informing NGOs’ own activities. Likewise, existing literature may be more significant when research aims to enhance wider knowledge than with research aiming to describe local conditions, where literature is often unavailable. Organisational principles also affect their approach. For example, Chalk’s consultation with government on proposed topics fits a commitment to “*work hand in hand with the key stakeholders, like government, with the relevant line ministries*” (programme coordinator), and their interest in research proposed by academics fits a stated commitment to academic partnerships.

While appropriate criteria and sources of ideas are likely to vary, these examples indicate that service delivery is not the NGOs’ only basis for identifying relevant research topics. Other criteria and sources are valued, such that some questions arising through service delivery are considered unsuitable and some questions that NGOs want to pursue do not come from service delivery.

### Identification of research questions by service delivery staff

The idea that NGOs can generate research questions through their service delivery suggests that service delivery staff can and do identify topics where research is needed. However, some case NGOs experience difficulty in eliciting research ideas from service delivery staff. Partly because of this, topics are often identified by senior managers and research staff rather than staff directly engaged in frontline delivery.

This is seen in Chalk. The research manager regularly emails staff, including service delivery managers, to request research ideas and seek feedback on possible topics. Few staff reply:

“[The research manager] *circulates an email, people respond, others don’t.*”

(interviewer) “*When you say people respond, does anyone respond?*”

“*Er…maybe…maybe less than 5%, I think the response is bad, I must just be frank. I’m not either good!*” (programme advisor)

This limited response to requests for research ideas reflects several interacting factors, including capacity and prioritisation among service delivery staff and the process used to gather research ideas.

#### Capacity to identify and propose research topics

In relation to capacity, one constraint is limited research experience. In Flint, few service delivery staff have designed or undertaken research, and senior managers feel this limits their ability to identify potential topics:

“[Research is] *a new department which is just coming in and people are yet to get into touch with it.* […] *We are trying to make efforts of how do we involve everybody in this. But that does not just come in a vacuum, you need to take people through a process, how do they identify research topics.*” (programme manager)

Lack of research experience or training makes it harder to understand the kind of issues that research could address or to conceptualise research questions.

In Chalk and Marl, staff are more familiar with research. Their experience of identifying research questions points to capacity constraints arising from service delivery involvement. One constraint is limited time to participate in decisions on future research. Service delivery staff are too busy running service delivery activities to respond to requests for research ideas:

“*Here at implementation level we’ve got so much on our table.* […] *A few hours you are in the office, a few hours you are there in the field seeing how things are, you are all over trying to make things work.* […] *So when this one comes* [requests for research topics]*, it’s just in the middle of doing something.*” (chalk programme manager)

While time to submit ideas is important, limited feedback from service delivery staff also reflects a lack of ideas for potential research. If staff were identifying research questions during their work, responding to emails about future topics would simply involve noting down their existing ideas. However, such ongoing identification of potential topics is hindered by their focus on daily service delivery. In particular, an emphasis on meeting plans agreed with donors and managers reduces time and space to think beyond immediate deliverables:

“*People like us, we are just interested in implementation because we have a project target to meet. So, we are just focused on meeting our targets and making sure things happen. So sometimes it’s difficult to generate research topics.*” (chalk programme manager)

The focus on delivery impedes critical reflection on current practice, such that service delivery staff do not consider research to assess programme effectiveness, or imagine alternative approaches that could be tested through research. Chalk staff criticised a culture of ‘business as usual’, meaning “*our programmes do not change much, and sometimes we adopt a traditional way of doing things*” (programme manager). The implication for development of research ideas was stated bluntly by another programme manager, who suggested:

“*The generation of research ideas or topics in our work. Are we doing business as usual? Or are we experimenting? And then the research ideas ought to come from that. How effective are we, or are NGOs, in terms of identifying research topics?*”

(interviewer) “*How effective are they*?”

“*I think not much*.”

This acceptance of current practice may be a particular issue for long-term staff who are socialised into organisational approaches. Many staff have worked at Chalk for over 10 years, and “*there’s certainly an element of ‘this is the way we’ve always done it and it works, sort of, so we’ll keep doing it’*” (programme manager). New staff can be better placed to identify research questions. Marl’s research officer emphasised the value of fresh insight when explaining that a new programme manager suggested one study:

“*She took this over, and of course when taking it over you realise, ‘OK, this is ongoing, but is it actually working or not?’ You ask these questions*.”

As well as identifying assumptions about current practice, new staff can bring ideas from their previous experience about alternative models to examine through research. For example, research to pilot a community care model was proposed by Marl’s research officer, who saw this approach in her previous job.

In addition to hindering reflection on current practice, the focus on immediate service delivery can limit attention to wider issues that might indicate research questions. Issues such as international debates about particular interventions or political structures that affect programme models have prompted research in Chalk, but raised largely by the research manager or senior directors. Service delivery staff “*tend to focus much more on the day to day activities, and it’s quite hard to get them to pull back and look at the more strategic importance of what they’re doing*” (chalk programme manager).

Exposure to discussions beyond immediate service delivery, through channels such as external networks, meetings or reading, can counter these constraints and so help staff identify questions. One way exposure helps is by creating awareness of alternative service delivery approaches or potential problems with current practice. For example, Chalk’s director explained that books he had read challenged aspects of their current model, contributing to his interest in research to test this and other service delivery approaches. Exposure also provides information on wider policy issues where research could contribute. In Marl, the senior managers and research staff who identify most research topics are more involved in external policy networks, enabling a “*broader viewpoint*” and “*idea of what else is going on in the country and outside of the country*” (research coordinator). This in turn helps these staff to identify research topics with wider relevance. Identifying such questions can be harder for frontline service delivery staff; Marl’s research coordinator compared their limited exposure to that of academics:

“*One of the nice things about academia is that you’re often going to conferences, you may have a meeting every week with your research group where people present different information. If you’re a field level implementer, you may never have that. You may be in the hospital every single day. Not everyone has access to all the different journals and things to have more this global picture.*”

Even the research coordinator struggled to identify potential research questions before attending meetings outside Marl:

“*I had a hard time myself even coming up with new ideas initially. And then* […] *they started sending me to more of these events where I got to see what other research was being done and why it was important and what gaps we had*.”

Exposure through external events, rather than involvement in service delivery, generated research ideas.

#### Low prioritisation of research

Identification of research topics is also affected by low prioritisation of research. Across the case NGOs, research is secondary to service delivery:

“*The challenge is always to see value for research in a development project. Because that is a software service, if you understand what I mean. You don’t immediately see the value added out of research*.” (chalk programme manager)

Benefits from research are considered less tangible than the ‘hardware’ of service delivery, and this means staff may not make time to consider possible research questions. Explaining the limited response to requests for research ideas in Chalk, a programme advisor admitted that “*it’s just the interest to engage in the process, I think just bluntly that’s it*”.

Organisational responsibility for research affects motivation to help identify research topics. Chalk staff tend to focus on work for which they are directly responsible, rather than engaging in activities led by others:

“[lack of feedback] *is a common phenomenon in Chalk and it’s not just the research issues. Most of the issues when they are circulated you rarely get comments from people.* […] *People are busy with their ‘own things’, in quotes.*” (programme manager)

Other staff see research as primarily the research manager’s responsibility, reducing their motivation to provide input. In contrast, in Marl, the service delivery director has overall responsibility for research, and consequently this director is fully involved in suggesting research topics. In one of the mini case NGOs, responsibility of service delivery staff for identifying topics is cemented by placing research funding within service delivery budgets. Service delivery managers outline their needs and the research team just help to refine ideas. By giving service delivery staff greater responsibility for research, these structures increase the priority of research within their workloads and their role in identifying questions.

#### The process used to identify research ideas

For service delivery staff to suggest research topics, the process used to gather ideas must accommodate staff capacity and motivation. Chalk’s use of email to request research topics does not fit the hectic workloads, limited ongoing reflection and low prioritisation of research. With this approach, “*there isn’t much room to think about it*” (project manager). An alternative approach is seen in Marl, where training workshops have helped service delivery staff to identify research questions. Although frontline staff rarely suggest topics for Marl’s larger research studies, these staff have planned and undertaken small research projects on the activities they manage. Training focuses on research skills, but plausibly counters other obstacles to development of research ideas by providing time, motivation and an opportunity for dialogue that enables reflection on current practice.

The constraints of capacity and motivation within the case NGOs do not mean service delivery staff never suggest research questions or that research questions are unrelated to service delivery; the research staff and senior managers who tend to identify topics are familiar with service delivery concerns. However, limited input to research agendas from some service delivery staff suggests that close involvement in service delivery does not automatically stimulate research ideas.

### NGOs’ ability to pursue research questions they consider relevant

When the case NGOs identify research questions through their service delivery experience that they want to pursue, they are sometimes unable to take these questions forward; they face obligations to undertake research they see as low priority and constraints that prevent desired research being undertaken. Two related aspects that affect NGOs’ control over their research agenda are relationships with organisational headquarters or donors and financial position.

#### The influence of headquarters offices and donors on research agendas

Headquarters offices have a particular influence on research agendas in Marl. Sometimes, the headquarters propose topics, particularly for multi-country studies. Some proposed topics are considered relevant by Malawi country staff and the proposals are invitations that can be rejected; the research coordinator emphasised that they consider suggestions from the headquarters in relation to national priorities:

“*We always have to balance the needs of the country with the larger Marl objectives.* […] *Doing cross-country analyses can be very powerful, but it should not be at the expense of the country in which you’re currently in.*”

However, staff in Malawi appeared hesitant to reject headquarters proposals, even when topics are not their priority. Comments from the research officer about one study initiated by the headquarters indicated this perceived obligation: “*I didn’t even think it was so great, but we were asked to do it*”.

As well as proposing topics, Marl’s headquarters affect the research agenda through authorisation for topics suggested by the Malawi team. Staff in Malawi saw the approval process as reasonable – decisions are “*always open for discussion*” (research coordinator), and the process involves criteria that Malawi staff appreciate, including consideration of existing literature and international relevance. Most ideas from the Malawi team have been approved. However, this headquarters role means their research agenda is not “*anything we want, it has to be approved by our technical team*” (programme director).

The influence of Marl’s headquarters reflects their organisational structure. Marl’s headquarters approve and fund activities in Malawi. Chalk, the other international NGO, is more decentralised, putting control over the research agenda with Chalk’s Malawi office:

“*The bulk of resources, and I suppose the bulk of power really, sits within the Malawi programme. So our Malawi office is able to develop their own links with research institutions, their own research agenda, their own research priorities*.” (UK staff member)

This decentralisation increases the potential role of country-level service delivery experience in the research agenda.

While Marl’s financial and administrative structure means their headquarters influence research topics, donors are more significant in the other case NGOs because they depend heavily on donor funding. The extent of donor influence varies with different donor approaches and NGOs’ financial dependence. Sometimes, donors make particular studies mandatory: it is “*not that we are doing it as our programme, but probably we are supposed, we are expected to do that*” (former flint manager). These “*donor driven type of research projects*” (flint programme manager) are sometimes considered relevant by NGO staff, but NGOs have little choice of research topic if they want the donor’s funding. In more flexible projects, donors set a broad research agenda within which NGOs can propose topics. One example is research by Flint on counselling needs among young people. This research was undertaken as part of an NGO network project on reproductive health, and had “*to be within the broader aim on education, sexual and reproductive health and HIV and AIDS*”. However, “*as individual organisations, we were supposed to further explore and expand on what our ideas are going to be*” (former flint manager). The most flexible situations involved NGOs identifying topics and just consulting donors. This applies to research in Chalk funded by one long-term donor. The research manager and donor both emphasised that the donor does not tell Chalk what research to do. However, Chalk need to find topics of interest to the donor, and this constrains the agenda. This influence is evident in an email from the research manager to staff about potential research, which notes in relation to one topic that the donor “*may or may not go for it*” and says another topic “*may appeal to* [the donor]”. In all these situations, financial dependence on donors means research agendas are shaped by more than NGOs’ research priorities, and cannot simply respond to questions arising from service delivery.

#### Availability of funding for research

Closely linked with the influence of donors and headquarters, availability of funding affects which research questions are pursued. The effect of funding in determining research agendas varies sharply across the NGOs because of different financial situations. Flint depends almost entirely on donors and provides the clearest example of funding as a constraint. Several research ideas, some based on service delivery experience, were delayed or abandoned due to lack of funds. In contrast, Marl’s funding comes primarily from public donations, meaning they do not need to secure donor grants. The Malawi office receives funding from the headquarters based on annual plans that can include research and additional funds can be requested as needed. Although plans must receive headquarters approval, this internal funding is more secure than applying to donors and approval is likely, as explained in relation to the headquarters influence. This financial situation gives Marl considerable freedom to pursue the research they choose: “*In Marl we are very, very lucky to have that flexibility*” (programme director).

Beyond absolute availability of funds, the amounts available also affect which research questions are followed. In Chalk, some research funding is secured through a long-term grant, but the limited budget for each study affects topic selection. Some questions of interest to Chalk service delivery staff are judged impractical given available funds.

Combined with the influence of headquarters or donors, these financial constraints mean NGOs’ actual research agendas sometimes differ from their ideas about what research would be relevant, and questions arising from service delivery cannot always be translated into research projects.

### The perceived relevance of research findings

A final issue affecting the role of service delivery experience in producing relevant research is whether questions originating from service delivery lead to relevant research findings. The case NGOs’ experiences suggest there is no simple relationship between the source of research questions and perceived relevance of completed research.

This is partly because, as explained in the Background, relevance is a debated concept. Case NGO staff have a highly instrumental understanding of relevance, wanting research that “*feeds directly into our programme activities*” (marl research coordinator) and “*focuses on the work that we are doing, immediately*” (flint director). Research should “*address a felt knowledge gap*” (chalk programme coordinator) and “*end up with something that can be implemented*” (chalk programme advisor), either “*informing internal programming, or external advocacy*” (chalk programme advisor). In line with this approach, Flint’s research on counselling needs was considered relevant by staff because the findings were used to design training for community volunteers, such that “*there is a good a linkage in terms of the survey informing different stages of programming*” (flint programme manager). As described in the Background, some academics and civil society organisations contest this instrumental approach to relevance, arguing for research that addresses wider frameworks and discourses rather than immediate programmatic needs [42, 43]. Such different views on appropriate goals for research preclude any automatic link between questions based on service delivery and relevance.

Even accepting an instrumental definition of relevance, the case NGOs’ experiences suggest a question arising from service delivery experience does not guarantee relevant findings. One reason is that interests vary between staff and organisations, and “*relevance is subjective*” (chalk programme advisor). Research initiated by an NGO based on their service delivery and seen as relevant by some people may be considered irrelevant by other staff or partners. Varied ideas within NGOs are a particular issue for Chalk because their broad organisational agenda means staff have diverse areas of expertise, for example, HIV, nutrition or sanitation. Staff are sometimes unaware of research gaps in other areas, and see research on their own subject as more important. Expertise in specific areas also makes it harder to understand research on other topics, discouraging staff from reading research reports:

“*The moment an issue goes a bit more technical then you find that it’s only people that are conversant with that field that would feel comfortable.* […] *The moment people read two paragraphs and they can’t understand, well why bother?*” (programme manager)

This difficulty in understanding can mean staff dismiss studies as irrelevant without even knowing the findings.

Different views on relevance between NGOs and external partners are illustrated by Marl’s experience. Some Marl research is considered highly relevant by some external organisations. For example, a national government research advisor called Marl a “*centre of excellence*” for research and praised their reports. However, external interest varies between studies and audiences. Describing district government interest in research on a health intervention introduced by Marl, the research officer said there was:

“*Not much really, because it was so much Marl projects*. […] *Marl was paying for the clients, for the staff* […] *The district said from the beginning ‘we will not be able to take that over’, so their interest in any evaluation was minimal*.”

Although this research was based on Marl’s service delivery experience, it examined an intervention that the government could not afford, reflecting the long-standing issue of NGO projects as “*islands of excellence*” [46], and reducing perceived relevance among government partners.

The link between questions from service delivery and relevant findings is also affected by subsequent stages of the research process after identification of questions, including design, data collection, analysis and writing, and dissemination. The influence of design was seen in a study developed by Clay’s donors without consultation on detailed objectives or methods. Although Clay felt the topic was useful, the design involved “*tools that are not really addressing many issues*” (clay manager), leading to findings that did not meet their needs.

Weak data collection, analysis and report writing can also mean research does not deliver expected outcomes. With one Flint study, staff saw research questions as relevant, but due to “*substandard*” analysis and writing, “*when we looked at that report we said ‘wow, this report is not giving us anything*’” (flint programme manager). Quality of data collection and analysis also affects external perceptions of relevance. Some donors and government partners saw Clay’s participatory action research as valuable because they felt the open discussion with communities produced more reliable findings than surveys: “*Once you get to the community, you start familiarising yourself, you chat informally and so pick up much more information.* […] *This other type of research, you might not get 100% true responses*” (clay government partner). In contrast, concerns about inaccuracy contributed to a view among some academics that NGO research is rarely useful: “*When you look at the methods there are often loopholes.* […] *it’s not clear how they will manage the data and so you aren’t sure about the findings*” (academic).

Finally, perceived relevance also depends on dissemination approaches, in particular whether findings are discussed with staff to explain their value. In Chalk, internal dissemination relies largely on circulating research reports by email. Even when staff read the reports, they sometimes fail to understand why findings are useful or how they could be applied, particularly given varied expertise. Some staff suggested that disseminating findings through meetings might ensure everyone understands the implications, increasing each study’s perceived relevance.

## Discussion

Experiences from the case NGOs suggest relationships between service delivery experience and relevant research agendas are more varied and constrained than suggested by some of those calling for NGO involvement in research. Research questions are not only relevant when generated through service delivery and questions generated through service delivery are not always relevant; service delivery staff may lack capacity or motivation to identify research questions; NGOs may lack the control and funding to pursue research questions from service delivery; and questions generated through service delivery do not necessarily lead to relevant results.

On the first of these findings, the case NGOs do not see their own service delivery experience as the only relevant input to research agendas; they value other sources of ideas, such as external researchers or policy networks, and consider additional criteria such as existing literature or interest among partners. Service delivery is one source of research questions, but it is not always a sufficient or necessary basis for identifying topics that meet NGOs’ research aims. Consequently, recommendations that NGOs’ research questions must be based on their service delivery to be relevant [7] may be too restrictive; instead, service delivery experience should be considered alongside other sources and criteria. While not apparent in discussions of NGO research, this approach of considering multiple criteria and consulting different stakeholders follows guidelines on research prioritisation [4, 47, 48].

Second, involvement in service delivery is not enough to prompt research ideas, and can indeed limit capacity to suggest research questions. Service delivery staff may lack time to identify potential topics or discuss research agendas. They may be so focused on delivering activities or accustomed to organisational models that they do not envisage research to assess current service delivery or pilot alternatives, or consider wider discussions where research could contribute. Similar challenges are described in literature on NGO learning and innovation, which notes that hectic schedules, socialisation into organisational thinking and a focus on rigid delivery plans characterised by logframes and donor targets can constrain reflection on practice and development of new ideas [49–54].

While service delivery staff can identify research questions through their work, this requires conditions such as motivation, time, awareness of organisational assumptions and alternative approaches, knowledge of policy debates and existing literature, and familiarity with research. The potential absence of these conditions among service delivery staff is supported by experience from other NGOs, with literature noting gaps in time to conceptualise research [55], skills to define questions [7, 56], awareness of existing research [57], knowledge of wider policy [56], and in prioritisation of research [7, 56]. Supporting input from service delivery staff also requires an appropriate process to gather research ideas such as workshops or meetings that provide time and allow dialogue that enables reflection and creative thinking [50, 54].

Third, when service delivery does generate research questions that NGOs want to pursue, NGOs vary in their ability to follow these research priorities. Relationships with organisational headquarters or donors can mean NGOs’ research agendas include questions they see as unimportant, while insufficient funding sometimes means NGOs cannot pursue the questions they want. Research agendas in all organisations consider costs, but for NGOs that rely on donor funding, financial constraints mean greater compromise between research priorities and donor interests. This influence of donors on research agendas is reported for other NGOs [42, 58–60], and follows a wider pattern of donor influence on NGO activities [61–63], including in Malawi [64, 65]. Finally, research questions that are based on service delivery experience and seen as relevant by NGO staff do not necessarily produce relevant research findings. Priorities vary within NGOs and between organisations. Different interests mean any research, whether or not based on service delivery, is likely to be considered more relevant by some groups than others. The subjective nature of relevance is widely discussed in literature on the politics of research [33–36], but less clearly acknowledged in suggestions that NGOs can identify relevant questions. The perceived relevance of findings also depends on the research process, including design, data collection and analysis, and dissemination strategies. The impact of these later stages on relevance is recognised in the wider international health and development literature, including guidelines on research uptake [1, 66–68]. These processes mean relevance can be ‘lost in translation’ through inappropriate dissemination, as well as ‘lost before translation’ through choice of research topic [69].

While this article focuses on NGOs, the value of input from service delivery practitioners to research agendas is highlighted more broadly within the health research literature and guidelines on priority setting [4, 47, 48, 70–73]. WHO reports call for “*demand-driven research*” that tackles problems identified by policymakers and managers [13], and suggest that “*those on the front lines of policymaking or service delivery are particularly sensitive to what is working and what is not, and therefore they are able to point to important priorities for research*” [74]. While in no sense denying the instrumental and ethical value of involving practitioners in research prioritisation, the case NGO experiences suggest that practitioners may face difficulty in identifying research topics, and that they may welcome topics identified by researchers. Attention to demand may be more effective if it involves support for the practitioners asked to identify questions, and dialogue rather than a one-way flow of requests from policy or programme managers. Similar conclusions have been reached in the different context of United Kingdom health research, where experiences of research commissioning indicate difficulties for practitioners and policymakers in specifying research needs, and the value of shared discussion between researchers and research users to define questions [75, 76].

These findings are based on a small number of NGOs. The case NGOs had diverse organisational contexts, and literature on NGOs indicates similarity between significant characteristics of the case organisations and other NGOs, for example, donor relationships. This suggests the findings may apply more widely. However, further research would be needed to test and review the conclusions in other organisational contexts.

## Conclusions

This article examined the role of NGOs’ service delivery experience in supporting relevant research. The findings suggest that the value and feasibility of drawing on NGOs’ service delivery to develop research agendas varies with research aims and organisational contexts. Recognising different aims, potential difficulties and enabling conditions can help in the understanding of whether and how to draw on NGOs’ service delivery in identifying research questions. In particular, the findings suggest that ideas from service delivery should be considered alongside other criteria and other sources of research questions and that service delivery practitioners may need support to identify topics from their experience. Further, NGOs need adequate freedom and funds to pursue research agendas they see as relevant, and promoting relevance depends on steps throughout the research process. Some specific implications for NGOs, academic partners and donors that support NGO research are suggested below.

The feasibility and effectiveness of these recommendations will depend on particular organisational contexts and, in some cases, on wider institutional changes. For example, steps to encourage identification of research questions among service delivery staff, such as enabling exposure beyond service delivery and providing research training, may be more effective when accompanied by broader measures to support organisational learning. Promoting learning within NGOs is challenging due to the complexity of development as well as the constraints of time, socialisation and project frameworks noted previously [77]. However, there are documented strategies to support reflection and help staff recognise gaps in current practice or alternative approaches, such as providing senior leadership, space to discuss difficult issues, and time, resources and goals for learning within project plans [50, 54].

Wider institutional contexts also affect scope for academic partners to ask NGOs about their research priorities. There are practical barriers related to time and funding. For example, effective consultation on research questions often requires investment in meetings to develop open relationships and discuss ideas, but funding is often unavailable for this early stage of research design [78]. Career incentives may limit academics’ ability to follow NGO suggestions for research topics even when these are identified. For example, academic job security may depend on theoretical contributions, international relevance or a singular disciplinary focus, requirements that do not always align neatly to questions identified by NGO practitioners [39, 79]. Academics can also confront the same obligation to meet donor interests faced by NGOs, with research funding calls having pre-set agendas that limit opportunities to respond to NGO suggestions. Working with NGO staff to identify research questions may be more feasible within the context of long-term partnerships that provide time and trust to discuss potential topics and find relevant funding opportunities [78].

Donors, too, may face constraints in identifying and responding to NGOs’ research priorities. The immediate donors of national Malawian NGOs are often international NGOs that also face funding constraints and donor restrictions, reducing their ability to respond to local partners’ interests [64]. For large government donors, bureaucratic structures, central policy directives and a focus on efficiency can limit discussion with NGO partners to understand their priorities, while power inequalities can reduce NGOs’ confidence to put forward alternative ideas [80]. However, even where broad agendas are set from higher in the aid chain, examples described by the case NGOs as ‘donor driven’ suggest scope for greater consultation. For example, disagreements often related to the specific focus within a broadly agreed research topic, suggesting some flexibility to adapt the research to NGO interests.

While broader structures will affect possible roles for service delivery experience in research agendas, the following recommendations are proposed as modest steps for NGOs, academics and donors to consider in identifying research questions.

NGOs:

- Do not restrict identification of research questions to internal consideration of service delivery experience. External researchers may have useful ideas for possible topics. Consultation with other stakeholders and target audiences, such as government, can help ensure research questions have wider relevance and do not duplicate existing reports.
- To support relevance, consider processes at different stages of research, not just identification of questions. Relevance of findings depends on, for example, the specific research design, producing research of sufficient quality to meet research aims, and using dissemination approaches that explain the significance of findings. On the latter, sharing findings with staff through discussion rather than email can help them appreciate the implications.

NGOs and academics working with NGOs to develop research agendas:

- In seeking input to research agendas from service delivery staff, consider what support is required. Depending on existing capacities and motivation, the following steps may be useful:
  - Consider using discussion rather than relying on email to ask staff for ideas. Discussion might overcome barriers related to time, motivation and skills to identify possible topics.
  - Given that staff may find it difficult to formulate questions, try asking about problems they are encountering rather than research questions.
  - Exposure to wider discussions and experiences sometimes helps identification of research topics by challenging assumptions about current practice and supporting awareness of wider debates. Support exposure through, for example, meetings with other organisations or discussions of existing literature.
  - Service delivery staff may be unaware of existing research that could answer their questions, and consequently the topics they suggest might not be research gaps. Supporting staff familiarity with existing research can avoid duplicating existing research and help fill knowledge gaps. This support could draw on existing resources about evidence literacy, which provide guidelines for identifying and assessing relevant research [57, 81].
  - Research training (for example, using workshops and mentoring to plan and implement small studies) may help staff to identify research questions, by supporting appreciation of research, providing time to think about research, and assisting formulation of research questions.
  - Giving programme staff responsibility for identifying research topics may encourage their input. However, depending on existing skills, this may need balancing with support for capacity to identify appropriate topics.

Donors:

- To ensure that ideas from NGOs’ service delivery can be considered in their research agendas, ask NGO partners about their research priorities and discuss funding research on these topics.
